# ROS-Mediated Anti-Tumor Effect of Coptidis Rhizoma against Human Hepatocellular Carcinoma Hep3B Cells and Xenografts

**DOI:** 10.3390/ijms22094797

**Published:** 2021-04-30

**Authors:** So Young Kim, Cheol Park, Min Yeong Kim, Seon Yeong Ji, Hyun Hwangbo, Hyesook Lee, Su Hyun Hong, Min Ho Han, Jin-Woo Jeong, Gi-Young Kim, Chang-Gue Son, JaeHun Cheong, Yung Hyun Choi

**Affiliations:** 1 Anti-Aging Research Center, Dongeui University, Busan 47340, Korea; 14731@deu.ac.kr (S.Y.K.); ilytoo365@deu.ac.kr (M.Y.K.); 14602@deu.ac.kr (S.Y.J.); hbhyun2003@naver.com (H.H.); 14769@deu.ac.kr (H.L.); 2 Department of Biochemistry, College of Korean Medicine, Dong-eui University, Busan 47227, Korea; hongsh@deu.ac.kr; 3 Department of Molecular Biology, Pusan National University, Busan 46241, Korea; 4 Division of Basic Sciences, College of Liberal Studies, Dong-Eui University, Busan 47340, Korea; parkch@deu.ac.kr; 5 National Marine Biodiversity Institute of Korea, Seocheon 33662, Korea; mhhan@mabik.re.kr; 6 Nakdonggang National Institute of Biological Resources, Sangju 17104, Korea; jwjeong@nnibr.re.kr; 7 Department of Marine Life Sciences, School of Marine Biomedical Sciences, Jeju National University, Jeju 63243, Korea; immunkim@jejunu.ac.kr; 8 Institute of Bioscience & Integrative Medicine, Daejeon University, 176 split 75 Daedeokdae-ro Seo-gu, Daejeon 35235, Korea; ckson@dju.ac.kr

**Keywords:** apoptosis, autophagy, Coptidis Rhizoma, Hep3B cells, migration

## Abstract

Coptidis Rhizoma is the dried rhizome from the *Coptis chinensis* Franch. that has been shown to have a number of beneficial pharmacological properties including antioxidant, anti-inflammatory, and anti-cancer effects. However, the anti-cancer effects of Coptidis Rhizoma on hepatocellular carcinoma (HCC) remain unclear. In this study, we investigated the anti-cancer properties of Coptidis Rhizoma ethanol extract (CR) in HCC Hep3B cells and in a xenograft mouse model. Our results showed that the CR significantly inhibited cell growth and induced apoptosis in Hep3B cells through increased expression of Bcl-2 associated x-protein (Bax) and cleavage of poly-ADP ribose polymerase (PARP), reduced expression of Bcl-2, and activated caspases. CR also increased the generation of intracellular reactive oxygen species (ROS), which caused a loss of mitochondrial membrane potential (MMP, ΔΨm) and activation of the mitochondria-mediated intrinsic apoptosis pathway. Moreover, *N*-acetylcysteine (NAC), a ROS inhibitor, markedly blocked the effects of CR on apoptotic pathways. CR also induced the expression of light chain 3 (LC3)-I/II, a key autophagy regulator, whereas CR-mediated autophagy was significantly suppressed by NAC. In addition, pre-treatment with NAC perfectly attenuated the inhibition of cell invasion and migration of CR-stimulated Hep3B cells. Furthermore, oral administration of CR suppressed Hep3B tumor growth in xenograft mice without toxicity, alterations to body weight, or changes in hematological and biochemical profiles. Taken together, our findings suggest that CR has anti-tumor effects that result from ROS generation, and may be a potential pharmacological intervention for HCC.

## 1. Introduction

Hepatocellular carcinoma (HCC) is the most common primary liver malignancy and the second leading cause of cancer death [1,2]. The most potent risk factors for HCC include chronic hepatitis B, chronic hepatitis C, alcohol consumption, obesity, aflatoxin-contaminated foodstuffs, smoking, and diabetes [2,3]. Strategies for the treatment of HCC vary between the early and advanced stages [3]. Despite the variety of therapies available during each stage, HCC patients have a high recurrence rate and most endure serious side effects during treatment [4,5]. Therefore, more effective and safer adjuvant therapy is needed. Numerous studies have reported that traditional medicines represent not only potential anti-cancer treatments, but also have low toxicity and fewer side effects than current therapies [6,7]. Moreover, Liu et al. [6] noted that alkaloids extracted from certain traditional medicines can treat a variety of cancers including liver cancer, through apoptosis and autophagy induction as well as by inhibiting cell proliferation, migration, and invasion. Therefore, a variety of traditional medicines are expected to be potentially effective in the treatment of HCC based on previous studies.

Coptidis Rhizoma is the rhizome of *Coptis chinensis* Franch., *C. deltoidea* C. Y. Cheong et Hsiao, or C. *teeta* Wall. (Ranunculaceae), and is used mostly in Asian countries for the prevention and treatment of various diseases. Many studies have shown that Coptidis Rhizoma exhibits a variety of pharmacological activities including anti-inflammatory, antiviral, antidiabetic, and antitumor effects [8,9]. Notably, isoquinoline alkaloids, which are primary components of Coptidis Rhizoma, are reported to possess pharmacological benefits including anti-cancer effects in HCC [10,11]. Nevertheless, the anti-cancer effects of Coptidis Rhizoma on HCC are not well-defined and require further study. The aim of the present study was to investigate the efficacy of Coptidis Rhizoma ethanol extract (CR) on HCC tumor growth in Hep3B cells culture and a xenograft mouse model.

## 2. Results

### 2.1. CR Induces Caspase-Dependent Apoptosis in Hep3B Cells

**Figure 1:**
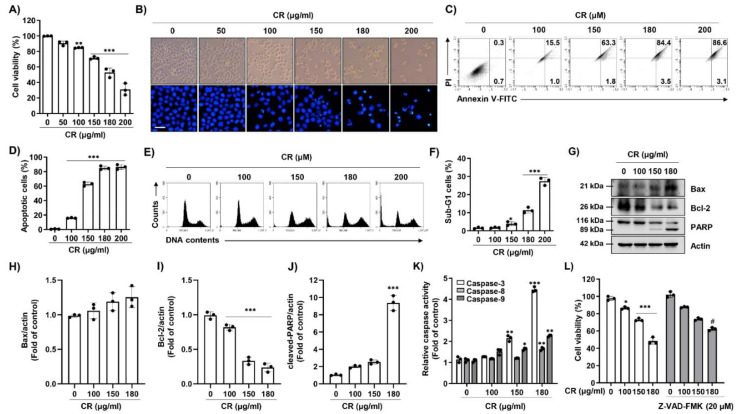
Coptidis Rhizoma ethanol extract (CR) promotes caspase-dependent apoptosis in Hep3B cells. Cells were treated with the indicated concentrations of CR for 24 h. (**A**) After 24 h, we added 0.5 mg/mL of 3-(4,5-dimethylthiazol-2-yl)-2,5-diphenyltetrazolium bromide (MTT) solution for 2 h at 37 °C. The data are expressed as mean ± standard deviation (SD) of three independent experiments. ** *p* < 0.01 and *** *p* < 0.001 when compared to control. (**B**, Top panel) Cell morphology was observed by a phase-contrast microscope at 50× magnification. (**B**, Bottom panel) After 24 h, cells were harvest and stained with 2.5 µg/mL of 4′,6′-diamidino-2-phenylindole (DAPI) solution for 20 min, and washed with PBS and then detected using a fluorescence microscope. Scale bars; 30 µm. (**C**) After treatment with CR for 24 h, cells were collected and stained with annexin V-fluorescein isothiocyanate (FITC) and propidium iodide (PI), and apoptosis-positive cells were measured by flow cytometry. (**D**) The frequencies of apoptosis-positive cells were quantified by expressing the number of annexin V^+^ cells (*n* = 3). *** *p* < 0.001 compared to control. (**E**) After 24 h of CR treatment, cells were collected and stained with PI and sub-G1 cells were detected by flow cytometry. (**F**) The apoptotic cells were quantified by expressing the sub-G1 cells (*n* = 3). * *p* < 0.05 and *** *p* < 0.001 compared to control. (**D** and **F**) The percentages of apoptotic cells were quantitated by expressing the numbers of annexin V^+^ cells and sub-G1 cells (*n* = 3). * *p* < 0.05 and *** *p* < 0.001 compared to control. (**G**) After treatment with CR for 24 h, cells were lysed, and equal amounts of proteins were analyzed using Western blot analysis to detect the expression of Bcl-2 associated x-protein (Bax), Bcl-2, and poly-ADP ribose polymerase (PARP). Actin was used as a loading control. (**H**–**J**) Bar graphs indicate the relative band density in western blot analysis (*n* = 3). *** *p* < 0.001 compared to control. (**K**) After 24 h, cells were harvested and incubated with caspase-3, -8, and -9 substrates. The activities were measured by an enzyme-linked immunosorbent assay (ELISA) reader (*n* = 3). * *p* < 0.05, ** *p* < 0.01, and *** *p* < 0.001 compared to control. (**L**) Cells were pre-treated with or without 20 µM of benzyloxycarbonyl-Val-Ala-Asp (OMe) fluoromethylketone (Z-VAD-FMK) for 1 h, and treated with CR for 24 h. Cells were treated with MTT solution for 2 h at 37 °C. Cell viability was measured by the MTT assay (*n* = 3). * *p* < 0.05 and *** *p* < 0.001 compared to control, and # *p* < 0.05 compared to CR-treated cells.

To investigate the effect of CR on Hep3B cell proliferation, the cells were exposed to various concentrations of CR for 24 h and cell viability was measured by a 3-(4,5-dimethylthiazol-2-yl)-2,5-diphenyltetrazolium bromide (MTT) assay. Figure 1A shows that CR significantly suppressed cell viability in a dose-dependent manner. Additionally, we investigated cytotoxicity effect of CR on several cell lines such as mouse macrophage RAW 264.7 cells, human keratinocyte HaCaT cells, normal Chang liver cells, human lung cancer A549 cells, human hepatoma HepG2 cells and Huh7 cells. CR had more cytotoxicity on the p53 mutant carcinoma cells, including Hep3B cells and Hur7 cells, than p53 wild carcinoma A549 and HepG2 cells (Supplementary Figure S1). Furthermore, CR appeared more strongly sensitivity in carcinoma cells compared normal cells. At the same time, cell density decreased, the number of detached cells increased, and morphological modifications such as shrinkage and irregular cell surface were identified (Figure 1B). To determine whether CR-induced cell growth inhibition was related to apoptosis, we observed nuclear morphology using 4′,6′-diamidino-2-phenylindole (DAPI) staining. As shown in the lower panels of Figure 1B, morphological changes such as nuclear fragmentation and chromatin condensation were observed in CR-treated cells. In addition, annexin-V/propidium iodide (PI) staining showed a CR dose-dependent increase in apoptotic cells (Figure 1C,D) as well as in the percentage of sub-G1 cells (Figure 1E,F). Later experiments excepted a concentration of 200 µg/mL CR because it caused serious damage and decreased density in cells. In order to determine whether apoptosis-related genes were modulated by CR treatment, Western blot analysis and caspase activity assays were performed. As shown in Figure 1G–J, CR upregulated the expression of pro-apoptotic Bcl-2 associated x-protein (Bax) and poly-ADP ribose polymerase (PARP) cleavage, and downregulated the expression of anti-apoptotic Bcl-2. In the caspase activity assays, CR dose-dependently activated caspase-9 and -3, while the activity of caspase-8 was only slightly increased at the highest concentration (Figure 1K). To confirm the effect of caspase on CR-induced apoptosis, we used benzyloxycarbonyl-Val-Ala-Asp (OMe) fluoromethylketone (Z-VAD-FMK), a pan-caspase inhibitor. Figure 1L demonstrates that pre-treatment with Z-VAD-FMK blocked the CR-mediated inhibition of cell growth. These results suggest that CR induced apoptosis through the caspase-dependent pathway in Hep3B cells.

### 2.2. CR-Induced Apoptosis Is Associated with ROS-Mediated Mitochondrial Dysfunction in Hep3B Cells

**Figure 2:**
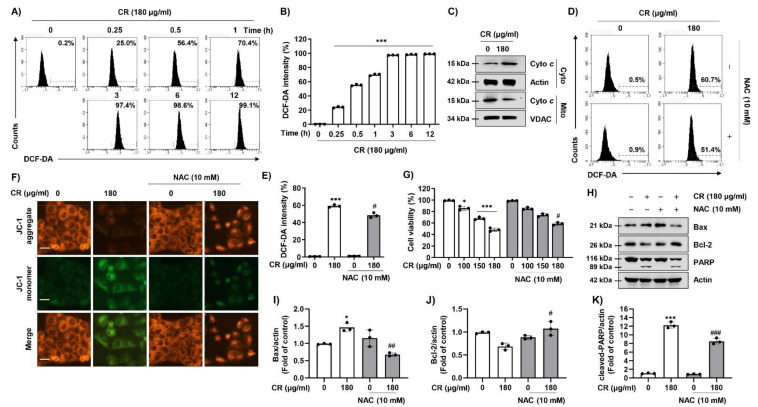
ROS regulates CR-mediated apoptosis in Hep3B cells. (**A**) Cells were treated with 180 µg/mL CR for the indicated times and cells were treated with 2′,7′-dichlorofluorescein diacetate (DCF-DA) staining solution for 20 min at 37 °C. Cells were washed with PBS and collected. The levels of ROS were measured by a flow cytometer. (**B**) The levels of ROS were presented as a quantification of fluorescence intensity (*n* = 3). *** *p* < 0.001 compared to control. (**C**) Cells were treated with 180 µg/mL CR for 24 h and harvested and then mitochondrial and cytosolic proteins were isolated by a mitochondria isolation kit. The equal amounts of proteins were performed Western blot analysis to detect. Actin was used as a loading control. (**D**,**E**) Cells were pre-treated with or without 10 mM of *N*-acetylcysteine (NAC) for 1 h, and then cells were treated with 180 µg/mL CR. After 1 h, cells were stained with DCF-DA for 20 min and measured ROS levels, quantitated using a flow cytometer. Data are expressed as the mean ± SD (*n* = 3). *** *p* < 0.001 compared to control, and # *p* < 0.05 compared to CR-treated cells. (**F**–**H**) After 24 h of CR treatment, cells were incubated with of 5,5′6,6′-tetrachloro-1,1′,3,3′-tetraethyl-imidacarbocyanine iodide dye (JC-1) for 20 min, and the fluorescence intensity of JC-1 was observed with a fluorescence microscope. Scale bars; 30 µm. (**G**) After 24 h of CR treatment, the cell viability was measured by the MTT assay. Data are expressed as the mean ± SD (*n* = 3). * *p* < 0.05 and *** *p* < 0.001 compared to control, and # *p* < 0.05 compared to CR-treated cells. (**H**) Cells were pre-treated with or without NAC for 1 h and then treated with 180 µg/mL of CR for 24 h. Cells were lysed, and equal amounts of proteins were analyzed using Western blot analysis. The expression of apoptosis-related proteins (Bax, Bcl-2, and PARP) were detected by Western blotting. Actin was used as a loading control. (**I**–**K**) Bar graphs indicate the relative band density in Western blot analysis (*n* = 3). * *p* < 0.05 and *** *p* < 0.001 compared to control; # *p* < 0.05, ## *p* < 0.01, and ### *p* < 0.001 compared to CR-treated cells.

Based on the upregulation of Bax and downregulation of Bcl-2 expression by CR treatment, we investigated whether CR-induced apoptosis was associated with mitochondrial function. Since excess intracellular ROS can cause mitochondrial dysfunction, we assessed the effect of CR on the generation of ROS. As shown in Figure 2A,B, intracellular ROS levels peaked after 3 h following CR treatment, and that increase was maintained out to 12 h. In addition, CR significantly induced cytochrome *c* release mitochondria into the cytosol (Figure 2C). However, CR-induced intracellular ROS production was significantly suppressed by *N*-acetylcysteine (NAC), a ROS scavenger (Figure 2D,E). In this regard, NAC also suppressed the upregulated expression of the 5,5′6,6′-tetrachloro-1,1′,3,3′-tetraethyl-imidacarbocyanine iodide dye (JC-1) monomer considered to cause loss of mitochondrial membrane potential (MMP, ΔΨm) in CR-stimulated cells (Figure 2F). Furthermore, NAC not only improved cell viability, but also substantially reversed the changes to expression levels of Bax, Bcl-2, and PARP following CR treatment (Figure 2E–K). These findings demonstrate that CR-mediated apoptosis was associated with mitochondrial dysfunction via ROS production in Hep3B cells.

### 2.3. CR Induces ROS-Mediated Autophagy in Hep3B Cells

**Figure 3:**
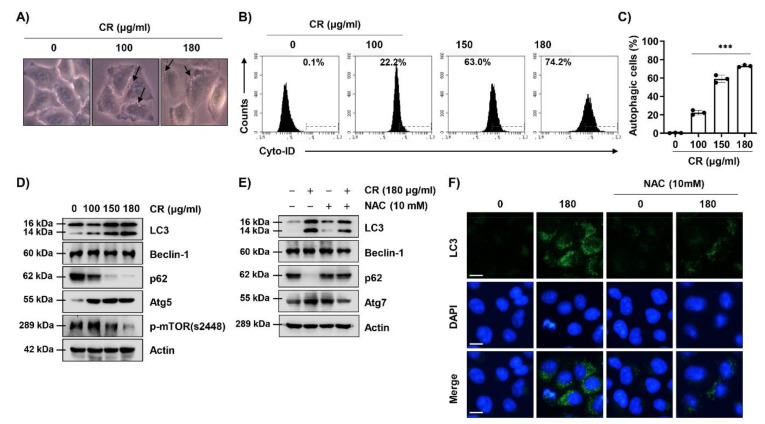
CR induces autophagy in Hep3B cells. (**A**–**D**) Cells were treated with the indicated concentrations of CR for 24 h. (**A**) Autophagic vacuoles were observed using a phase-contrast microscope at 400× magnification. Arrows present autophagic vacuoles. (**B**,**C**) After treated with CR for 24 h, cells were collected and incubated with Cyto-ID staining solution for 30 min, and autophagic cells were measured and quantitated by a flow cytometer (*n* = 3). *** *p* < 0.001 when compared to control. (**E**,**F**) Cells were pre-treated with or without 10 mM of NAC for 1 h, and then cells were treated with 180 µg/mL of CR for 24 h. (**D**,**E**) Cells were harvested and lysed. The equal amounts of proteins were analyzed using Western blot analysis. The expression of microtubule-associated protein 1 light chain 3 (LC3)-I/II, p62, autophagy related (Atg) 5, Atg7, Beclin-1, and phosphorylated mechanistic target of rapamycin (p-mTOR) was observed. Actin was used as a loading control. (**F**) The LC3 puncta (green) was detected by immunofluorescence and photographed using a fluorescence microscope. DAPI was used to counterstained the nuclei (blue). Scale bars; 15 µm.

To examine the effect of CR on autophagy induction, we observed the cell morphology and investigated the levels of autophagy. Figure 3A shows that autophagic vacuoles were observed in the cytoplasm of cells following exposure to CR. To examine autophagic compartments with minimal staining of lysosomes, we used Cyto-ID. Cyto-ID staining showed that CR dramatically induced Cyto-ID-positive autophagic cells in a dose-dependent manner (Figure 3B,C). In addition, the expression of microtubule-associated protein 1 light chain 3 (LC3) and autophagy-related (Atg)5, and Atg7, a key element of autophagy, was gradually increased, and p62 and phosphorylated mechanistic targets of rapamycin (p-mTOR) were decreased with increasing CR concentration. However, Beclin-1 was not altered by CR treatment. This regulation was blocked by NAC treatment (Figure 3D,E and Supplementary Figure S2). Similarly, the fluorescence expression of LC3 puncta corresponded with the Western blot analysis (Figure 3F and Supplementary Figure S2J). These results suggested that ROS production was involved in CR-induced autophagy in Hep3B cells.

### 2.4. CR Suppresses Cell Migration via ROS Production in Hep3B Cells

**Figure 4:**
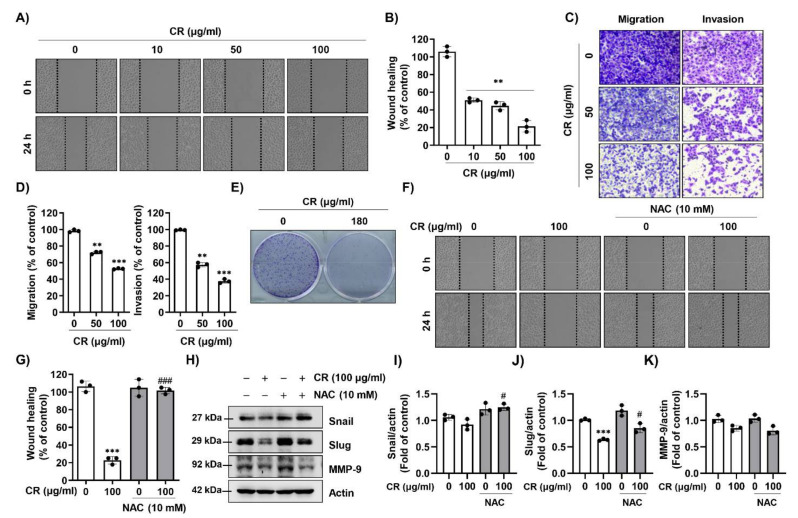
CR inhibits cell migration via ROS in Hep3B cells. (**A**) Cells were seeded, scratched, and treated with media contained 5% FBS and various concentrations (0, 10, 50, 100 µg/mL) of CR for 24 h. The migration of cell was measured with a wound healing assay (magnification; 50×). (**B**) Mobility was calculated, as compared to the control cells. Each bar represents the mean ± SD of three independent experiments. ** *p* < 0.01 when compared to control. (**C**) Cell migration and invasion assays were assessed using a trans-well chamber system. Cells were seeded in the upper chamber with serum-free medium containing CR and complement medium was added in the lower chamber. After 24 h of CR treatment, cells were stained with 0.1% crystal violet, and observed by a phase-contrast microscope at 100× magnification. (**D**) The numbers of migrating and invading cells were calculated, as compared with the control cells (*n* = 3). ** *p* < 0.01 and *** *p* < 0.001 when compared to control. (**E**) Hep3B cells were exposed to CR for 7 days, followed by colony formation assay. Cells were stained with 0.5% crystal violet solution, and visualized colonies were observed under a phase-contrast microscope. (**F**) Cells were pre-treated with or without NAC for 1 h, and treated with the CR for 24 h, and then cell migration was confirmed using a wound healing assay (magnification; 50×). (**G**) Mobility was estimated, as compared with the control cells (*n* = 3). *** *p* < 0.001 compared to control, and ### *p* < 0.001 compared to CR-treated cells. (**H**–**K**) After treatment with CR for 24 h, cells were lysed and equal amounts of proteins were analyzed using Western blot analysis to detect the expression of Snail, Slug, and matrix metalloproteinase-9 (MMP-9). Actin was used as a loading control. (**K**) Bar graphs indicate the relative band density in western blot analysis (*n* = 3). *** *p* < 0.001 compared to control; # *p* < 0.05 compared to CR-treated cells.

In order to determine whether CR can regulate the migratory and invasive ability of Hep3B cells, we performed a scratch wound healing assay and trans-well invasion assay. In the scratch wound healing assay, CR significantly decreased migratory ability compared to controls (Figure 4A,B). In addition, CR also markedly suppressed the cell migration and invasion ability of Hep3B cells (Figure 4C,D). Furthermore, we found that the colony forming ability of Hep3B cells was markedly decreased by CR relative to the control (Figure 4E). Next, we investigated whether the inhibitory effect of CR on cell metastatic activity was associated with ROS generation. Figure 4F,G shows that pre-treatment with NAC perfectly reversed the CR-mediated inhibition of migratory ability. In addition, CR decreased the expression of migration-related proteins such as Snail, Slug, and matrix metalloproteinase-9 (MMP-9). This inhibition was restored by NAC treatment (Figure 4H–K). These results suggest that CR inhibits migration and invasion ability of Hep3B cells via ROS regulation.

### 2.5. CR Inhibits Tumor Growth in the Hep3B Xenograft Model

**Figure 5:**
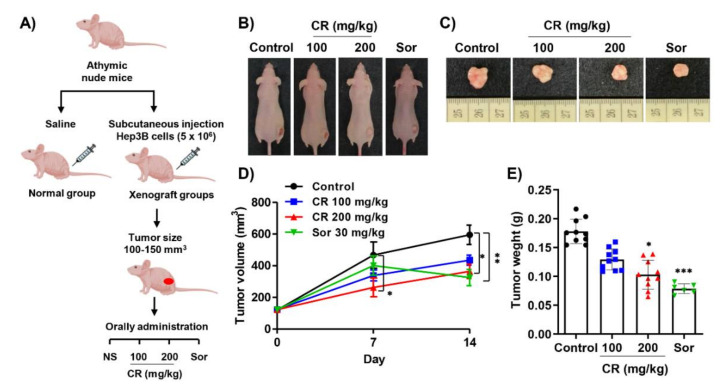
CR inhibits tumor growth in Hep3B xenograft model. (**A**) Experimental design of Hep3B xenograft model. The xenograft was induced in athymic nude mice by subcutaneous injected into the flank with 5 × 10^6^ cells/100 µL. Ten days after tumor injection, mice were randomly divided into four groups: Control (distilled water), low and high concentration of CR (100 and 200 mg/kg), and sorafenib (30 mg/kg). All treatments were orally administrated once per day for 14 days. (**B**) Image of the xenograft model showing the size of tumors treated with CR or sorafenib. (**C**) Representative images of the xenograft tumors. (**D**) Tumor volumes obtained from xenograft mice were measured once a week. Data presented as the mean ± SD (*n* = 6~10). * *p* < 0.05 and ** *p* < 0.01 compared to the non-treated group. (**E**) Tumors isolated from each group of xenograft mice were measured. Data presented as the mean ± SD (*n* = 6~10). * *p* < 0.05 and *** *p* < 0.001 compared to the non-treated group. (Sor; sorafenib).

We next investigated whether CR can suppress the growth of Hep3B xenograft tumors. Xenografted mice were divided into four groups as indicated in Figure 5A. During the experimental period, no subjects were lost and there were no differences in body weights between groups (data not shown). As shown in Figure 5B,C, administration of CR as well as sorafenib suppressed the growth of xenografted tumors compared to controls. In addition, the drugs (CR and sorafenib) led to significantly reduced tumor volume and weight when compared to the control group (Figure 5D,E). Organ weights including heart, lung, liver, kidney, and spleen were not different between groups (Supplementary Table S1). Furthermore, the results of hematologic and biochemical analysis also displayed no difference between groups (Supplementary Table S2). Thus, CR inhibited the growth of Hep3B xenograft tumors without toxicity, alteration of body weight, or changes in hematological and biochemical profiles.

### 2.6. Immunohistology in Hep3B Xenograft Tumor Sections

**Figure 6:**
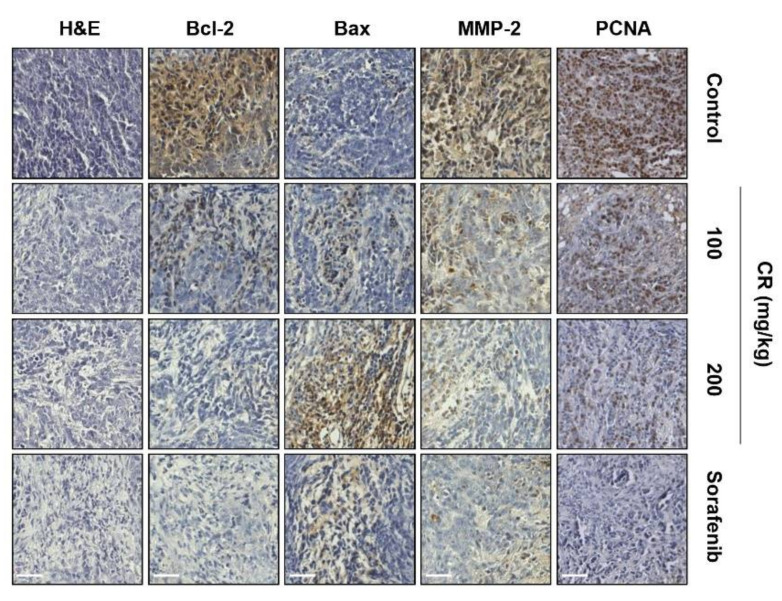
Immunohistology in Hep3B xenograft tumor sections in orally administration of CR. Tumor tissues were collected and fixed in 4% formaldehyde and embedded in paraffin blocks. Sections of 5 µm thickness were cut by microtome. Hematoxylin and eosin (H&E), Bcl-2, Bax, matrix metalloproteinase (MMP)-2, and proliferating cell nuclear antigen (PCNA) staining of tumor sections isolated from mice administered different treatments or controls. Scale bars; 40 µm.

We also confirmed the effect of CR on the histological alteration of xenograft tumor tissues obtained from CR or sorafenib treated subjects as compared to controls. Hematoxylin and eosin (H&E) staining showed that CR administration decreased the number of nuclei, although sorafenib appeared to do the same but with higher activity. In addition, both treatments created a scattered appearance with spaces evident between the cells, compared to the rather more uniform control group (Figure 6). To evaluate whether CR affected the proliferation and migration in xenograft tumors, we performed immunohistochemical analysis on the tumor sections. The results showed that expression of Bcl-2, matrix metalloproteinase-2 (MMP-2), and proliferating cell nuclear antigen (PCNA) were inhibited by both CR and sorafenib, while the expression of Bax was increased. These results demonstrate that CR might suppress tumor growth through the inhibition of enzymes that regulate migration and stimulate apoptosis.

### 2.7. Fingerprint Analysis of CR

**Figure 7:**
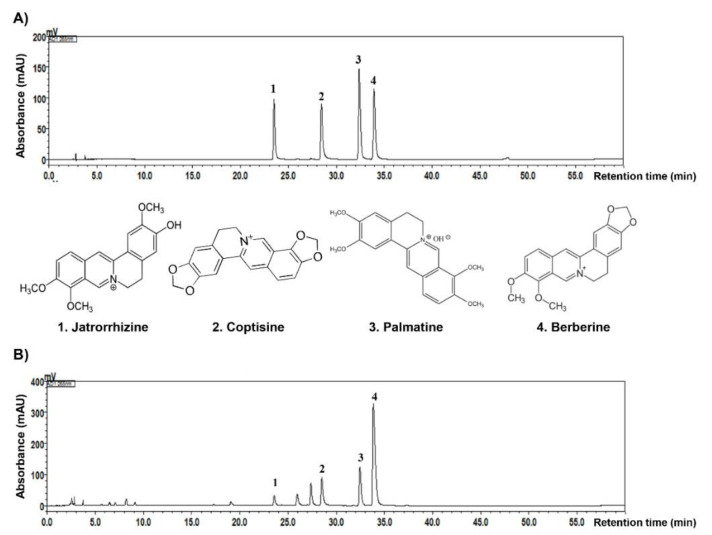
The natural compound fingerprint analysis from high-performance liquid chromatography (HPLC) of CR. (**A**) Four standard compounds of jatrorrhizine, coptisine, palmatine, and berberine were analyzed by HPLC. (**B**) Histogram from the HPLC analysis of CR.

Finally, to identify the bioactive compounds in CR we performed high-performance liquid chromatography (HPLC) analysis. Histograms of CR showed four major compounds (Figure 7A,B). The quantitation of CR was monitored at 265 nm. Results for each compound in the CR were in line with the standard; retention time was as follows: Jatrorrhizine (23.53 min), coptisine (28.38 min), palmatine (32.49 min), and berberine (33.89 min). The proportion of berberine was higher than other compounds, whereas coptisine and palmatine were present at similar levels.

## 3. Discussion

Apoptosis, otherwise known as programmed cell death, plays an important role as a homeostatic mechanism to maintain the balance of cell populations [12,13]. Apoptosis can be divided into extrinsic and intrinsic pathways. The extrinsic pathway begins when death ligands bind to DRs, such as tumor necrosis factor receptor-associated death domain and Fas-associated death domain, followed by caspase-8 activation [14,15]. The initiation of the intrinsic pathway is associated with mitochondrial dysfunction and Bcl-2 family proteins in response to various apoptotic stimuli. Thereafter, caspase-9 becomes activated and cytochrome *c* are released from the mitochondria into the cytoplasm [12,14]. Activated caspase-8 and -9 in turn activate caspase-3 and -7, which complete apoptosis by cleaving substrates such as PARP [12,13]. In this study, we investigated whether CR induces apoptosis in HCC Hep3B cells. Our findings showed that CR inhibited cell proliferation and induced apoptosis. The induction of apoptosis in Hep3B cells by CR involved the regulation of Bcl-2 family proteins and activation of caspases. In general, the mitochondria-mediated intrinsic apoptosis pathway is tightly related and results in increased production of ROS due to oxidative stress and loss of MMP (Δψm) [16,17]. Therefore, we investigated whether CR-mediated cell death is associated with ROS generation and mitochondrial dysfunction. Our results show that CR results in significant accumulation of ROS and consequent loss of MMP (Δψm). The role of ROS was confirmed by pre-treatment with the ROS scavenger NAC. Therefore, these data suggest that the induction of apoptosis by CR is related specifically to the mitochondria-mediated intrinsic pathway and occurs due to the production of ROS.

Autophagy is a well-known cellular stress response that facilitates the elimination of damaged organelles such as mitochondria and endoplasmic reticulum by lysosomes [18]. Although autophagy is commonly known to affect cell survival, it also promotes cell death in cancer therapy, including in HCC, and its role is supported by several studies showing that autophagy can be upregulated in response to anti-cancer therapeutics [19,20]. Moreover, many herbal medicines are known to have anti-cancer effects by regulating autophagy-mediated cell death in HCC cells [21,22]. In addition, autophagy is linked to ROS production and has two aspects: Tumorigenesis and tumor suppression [23]. Nonetheless, many chemotherapeutic drugs induce autophagy-mediated cell death through ROS production in HCC cells [24]. Herein, we found that CR induced autophagy in Hep3B cells, but was blocked by NAC pre-treatment. Although additional studies are needed to investigate the transcription levels of autophagy-related genes, our data show that the increase in ROS production by CR is likely responsible for the induction of autophagy.

The process of metastasis is a major therapeutic target as it plays a crucial role in cancer progression. Importantly, oxidative stress is well-known to promote metastasis, including in HCC cells [25,26]. In previous studies, various natural products or herbal extracts have demonstrated an ability to inhibit the metastasis of HCC cells without cytotoxicity [27,28]. These phenomena were associated with inactivation of MMPs or activation of tissue inhibitors including metalloproteinases-2 and E-cadherin [19,29]. In this study, we found that in Hep3B cells, CR inhibits wound healing, migration, and invasion, which experimentally are indicators of metastasis. However, the anti-metastatic potential of CR was completely blocked by the artificial inhibition of ROS production, indicating that the CR inhibition of metastasis involved ROS production. Furthermore, our results are in agreement with previous studies showing that some natural products with anti-cancer activity exert an ability to inhibit metastasis, which is dependent on autophagy induction and ROS production [23,28].

The anti-tumor efficacy of CR was identified in vivo by subcutaneously injecting HCC Hep3B cells into athymic nude mice. According to our results, both tumor volumes and weights were markedly suppressed by CR treatment in a dose-dependent manner, without any accompanying loss of body weight. Moreover, the morphological changes and expression of Bcl-2 family proteins, MMP-2, and PCNA in tumor tissues were regulated by CR treatment in vivo, and were consistent with the in vitro results. We also investigated biological parameters to investigate the toxicity of CR to the liver and kidneys. Alanine aminotransferase (ALT), aspartate aminotransferase (AST), and alkaline phosphatase (ALP) levels are established indicators of liver damage, while creatinine and blood urea nitrogen (BUN) levels are indicators of kidney function. There were no significant differences in the level of these enzymes or metabolites between CR- and sorafenib-treated xenograft mice compared to control mice. Therefore, our findings imply that CR inhibits the growth of Hep3B xenografted tumors without toxicity, weight changes, hematologic and biochemical profile changes. Our results align well with previous in vivo results from Iizuka et al. [30,31], showing that CR has anti-tumor activity without exhibiting signs of significant toxicity.

Major phytochemicals with anticancer activity contained in Coptidis Rhizoma include isoquinoline alkaloids such as berberine, coptisine, and palmatine [10,11], and it was confirmed by HPLC analysis that the CR used in this study also contained these compounds. Although there have been reports that these alkaloids inhibit the proliferation of HCC cells and promote apoptosis [32,33], there has been no investigation of the anti-cancer activity of the Coptidis Rhizoma extract itself against HCC cells. Therefore, our results support the results of previous studies demonstrating the anti-cancer activity of Coptidis Rhizoma against HCC.

In summary, CR inhibited cell proliferation and induced apoptosis through activation of the mitochondria-mediated intrinsic apoptosis pathway in HCC Hep3B cells. CR also promoted the induction of autophagy and suppression of cell migration and invasion. In this process, ROS could play as a potential target of CR-mediated inducing autophagy and decreasing migration, as well as partially influence mitochondrial-mediated apoptosis. Furthermore, CR inhibited tumor growth in a dose-dependent manner without toxicity in vivo. Although further studies are required to identify the role of p53 on the underlying mechanism of CR and to evaluate the efficacy of an active single compound derived from CR on HCC, our results provide strong evidence that CR represents a potential therapeutic for HCC treatment.

## 4. Materials and Methods

### 4.1. Preparation of an Ethanol Extract of Coptidis Rhizoma

Coptidis Rhizoma was obtained from the Dong-Eui Korean Hospital of Dong-Eui University (Busan, Republic of Korea) and was refluxed with 1 L of 70% ethanol solution by sonication for 24 h, as previously described [34]. After filtering, the extract was concentrated with a rotary vacuum evaporator (Buchi Labortechnik, Flawil, Switzerland), followed by freezing-drying, and then stored at −80 °C. The extract (CR) was dissolved in dimethyl sulfoxide (DMSO; Sigma–Aldrich Chemical Co., St. Louis, MO, USA) to a final concentration of 200 mg/mL. This stock solution was diluted to the required concentrations in culture medium prior to use. The control solution contained 0.05~0.1% (*v*/*v*) DMSO in a culture medium.

### 4.2. Cell Culture

Human HCC line (Hep3B, HepG2, and Huh 7) cells, human normal liver cell lines (Chang liver), mouse macrophages (RAW 264.7), human keratinocytes (HaCaT), and human lung cancer cell lines (A549) were purchased from the American Type Culture Collection (ATCC; Manassas, VA, USA). Cells were cultured with Dulbecco’s modified eagle’s medium (DMEM) or Roswell Park Memorial Institute 1640 medium supplemented with 10% fetal bovine serum (FBS), 2 mM of L-glutamine, 100 U/mL of penicillin, and 100 µg/mL of streptomycin (WELGENE Inc., Gyeongsan, Korea) at 37 °C in a humidified 5% CO_2_ atmosphere.

### 4.3. Cell Viability Assay

The effect of CR on Hep3B cell proliferation was determined using the MTT assay as previously described [35]. In brief, Hep3B cells were seeded into 6-well plates (1.3 × 10^5^ cells/well), treated with varying concentrations of CR for 24 h, and then had 200 µL of MTT solution (0.5 mg/mL, Sigma–Aldrich Chemical Co.) added at 37 °C for 2 h. After 2 h, the medium was removed and DMSO was added to dissolve the formazan for 10 min. Cell viability was measured at 540 nm using an enzyme-linked immunosorbent assay (ELISA) reader (VERSA Max, Molecular Device Co., Sunnyvale, CA, USA).

### 4.4. Cell Apoptosis Analysis

To determine apoptotic cell death, annexin-V-positive cells and the sub-G1 phase were detected by a flow cytometer. In brief, Hep3B cells were seeded into 6-well plates at a density of 1.25 × 10^5^ cells per well and incubated at 37 °C for 24 h. The cells were treated with CR for 24 h and collected before being stained with annexin V-FITC (BD Biosciences, San Diego, CA, USA) and PI (BD Biosciences) solution. To identify the cell cycle distribution, cells were treated with CR for 24 h, then stained with PI solution for 30 min. The frequencies of annexin-V-positive cells and cells belonging to the sub-G1 phase, which indicate apoptosis, were measured using an Accuri C6 flow cytometer (BD Sciences, Franklin Lakes, NJ, USA). To assess alteration in nuclear morphology, cells were fixed with 4% paraformaldehyde for 10 min and stained with DAPI (Sigma–Aldrich Chemical Co.) solution for 15 min before detection by a fluorescence microscope (EVOS FL Auto 2 imaging system, Thermo Fisher Scientific, Waltham, MA, USA) in the Core Facility Center for Tissue Regeneration at Dong-eui University (Pusan, Korea).

### 4.5. Measurement of MMP (Δψm)

To measure MMP (Δψm), cells were stained with JC-1 (Sigma–Aldrich Chemical Co.), which is an MMP (Δψm) indicator. After treatment with CR, cells were collected and stained with JC-1 for 20 min. The green (JC-1 monomers) and red (JC-1 aggregates) fluorescence were analyzed by a fluorescence microscope (EVOS FL Auto 2 imaging system, Thermo Fisher Scientific).

### 4.6. Measurement of ROS Production

Hep3B cells were seeded into 6-well plates at 1.3 × 10^5^ cells per well and incubated for 24 h. As described previously [36], cells were treated with or without 10 mM NAC, a ROS scavenger, for 1 h and then CR was added. Cells were then stained with 10 μM of 2′,7′-dichlorofluorescein diacetate (DCF-DA; Sigma–Aldrich Chemical Co.) for 15 min and subsequently harvested before analysis using an Accuri C6 flow cytometer.

### 4.7. Caspase Activity

Caspase activities were determined by colorimetric assay kits (R&D Systems, Minneapolis, MN, USA) as previously described [37]. Hep3B cells were seeded in 100-mm dishes at 6.5 × 10^5^ cells per well and stabilized for 24 h, and then treated with CR for 24 h. The cells were collected and lysed in lysis buffer. The collected supernatants were incubated with the supplied reaction buffer at 37 °C for 1–2 h. The caspase activities were measured at 405 nm using an ELISA reader.

### 4.8. Western Blot Analysis

Cells were pre-treated with or without 10 mM NAC for 1 h, and then incubated with CR for 24 h. The cells were harvested and whole cell protein was extracted using lysis buffer containing 250 mM NaCl, 25 mM Tris-Cl (pH 7.5), 5 mM ethylenediaminetetraacetic acid (pH 8.0), 1% NP-40, 1 mM 4-(2-aminoethyl) benzenesulfonyl fluoride hydrochloride, 5 mM dithiothreitol, and protease inhibitors. The mitochondrial and cytosolic proteins were isolated using a commercial mitochondrial fractionation kit (Active Motif, Inc., Carlsbad, CA, USA) in accordance with manufacturer’s procedure. Proteins were separated by sodium-dodecyl sulfate-polyacrylamide gel electrophoresis and then transferred onto polyvinylidene fluoride membranes (Millipore, Bedford, MA, USA). After membranes were blocked with 5% bovine serum albumin (BSA; Sigma–Aldrich Chemical Co.) at room temperature for 1 h, the membranes were incubated with primary antibodies overnight at 4 °C. Primary antibodies used in this study were anti-Bax, anti-Bcl-2, anti-PARP, anti-actin (1:1000, all from Santa Cruz Biotechnology, Santa Cruz, CA, USA), and anti-LC3-I/II (1:1000, Cell Signaling Technology, Beverly, MA, USA). Subsequently, membranes were washed with phosphate-buffered saline (PBS) containing 0.1% Tween 20 (PBST) and were incubated with the corresponding secondary antibodies (1:1500, Santa Cruz Biotechnology) at room temperature for 2 h. The signals were detected by chemiluminescence (Thermo Fisher Scientific) and then photographed using a Fusion FX Image system (Vilber Lourmat, Torcy, France). The quantification of band was calculated using Image J analysis software (National Institutes of Health, Bethesda, MD, USA).

### 4.9. Immunofluorescence

Hep3B cells were seeded into a 4-well chamber slide (3.0 × 10^4^/well), treated with CR for 24 h, and fixed with methanol (100%) at −20 °C for 10 min. After washing with PBS, cells were blocked with 5% BSA at room temperature for 1 h. The cells were incubated with a specific LC3 antibody (1:100) in 2.5% BSA overnight at 4 °C. The cells were washed using PBS containing 0.1% Triton X-100 (TBST; Sigma–Aldrich Chemical Co.) and were incubated with the secondary antibody (1:200, Abcam, Cambridge, UK) for 2 h at room temperature. The expression of LC3 was detected using a fluorescence microscope (EVOS FL Auto 2 imaging system, Thermo Fisher Scientific). The quantification of LC3 puncta was calculated using Image J analysis software.

### 4.10. Cyto-ID Staining

For the measurement of autophagy, Hep3B cells were seeded into 6-well plates and treated with CR for 24 h. As previously described [38], cells were collected, stained with Cyto-ID (Enzo Life Sciences, Inc., Farmingdale, NY, USA) for 30 min at room temperature, and analyzed using a flow cytometer.

### 4.11. Wound-Healing Assay

Hep3B cells were grown to 80–90% confluence in 6-well plates and scraped in a line with a 200-µL pipette tip. The cells were incubated with or without CR in 5% FBS-containing medium for 24 h. Images were captured using a phase-contrast microscope (Axio Scope, A1, Carl Zeiss, Oberkochen, Germany) and the relative width (%) of the scrape, representing wound healing, was compared between the initial scraping and after 24 h according to our published procedure [39].

### 4.12. Trans-Well Assay

Invasion and migration were assessed by the Trans-well chamber system (6.5 mm diameter, 8 μm pore size with polycarbonate membrane, Corning Costar Corp., Cambridge, MA, USA). The cells were placed in the upper chamber of the insert (5 × 10^4^/well) in serum-free DMEM with or without CR. For invasion assay, the upper chamber was pre-coated with Matrigel (BD Sciences). DMEM containing 10% FBS was added to the lower chamber. After 24 h, the upper chamber was stained with 0.5% crystal violet (Sigma–Aldrich Chemical Co.) for 30 min and washed with PBS. The invasion and migration activities were detected by a phase-contrast microscope (Axio Scope, A1, Carl Zeiss). The migration and invasion distances were calculated by Image J analysis software.

### 4.13. Colony Formation Assay

Hep3B cells were seeded in 6-well plates (1.3 *×* 10^5^ cells/well) and treated with 180 μg/mL of CR. After 24 h, cells were trypsinized and re-seeded in 6-well plates (1.5 *×* 10^3^ cells/well) with fresh medium and incubated at 37 °C. The cells were cultured for one week while replacing the medium every 3 days to form colonies. The colonies were fixed with 3.7% paraformaldehyde for 10 min and then stained with 0.1% crystal violet solution (Sigma–Aldrich Chemical Co.) at room temperature for 30 min. The stained colonies were observed and counted under a phase-contrast microscope and photographed.

### 4.14. Tumor Xenograft Experiment

All animal procedures were followed in accordance with the guide for the Institutional Animal Care and Use Committee of Dong-eui University (No. R2020-002). Fifty-one female athymic nude mice (4 weeks old) were purchased from KOATECH Laboratory Animals Inc. (Pyeongtaek, Korea). After 1 week to allow for adaptation, five mice were randomly assigned to the normal group and 36 mice were inoculated with Hep3B cell suspension (5 × 10^6^ cells) subcutaneously into the right flank as previously described [40]. When tumor volume reached 100–150 mm^3^ in the Hep3B cell-injected mice, the mice were randomly assigned to four groups: Controls (*n* = 10), a 100 mg/kg CR group (*n* = 10), a 200 mg/kg CR group (*n* = 10), and a 30 mg/kg sorafenib group (*n* = 6, Selleck Chemicals, Houston, TX, USA) as a positive control. Normal and control groups were administered distilled water. All of the treatments were administrated once a day for 14 days. Tumor volume and body weight were measured weekly. The formula for the tumor volume was as follows: Tumor/volume (mm^3^) = (w^2^ × l)/2 [w; width, l; length]. At the end of the experiments, all animals were sacrificed using CO_2_ and blood was collected for hematological and biochemical analysis. In addition, tumors were surgically isolated and fixed in 4% formaldehyde for further histomorphological analysis as previously described [41].

### 4.15. Immunohistochemistry

Tumor tissues were collected and fixed in 4% formaldehyde and embedded in paraffin blocks. Sections of 5-µm thickness were cut by microtome (Leica RM2125, Leica Biosystems, Heidelberg, Germany). For H&E staining, the sections were deparaffinized before being stained with H&E. For immunohistochemistry, after being deparaffinized, the sections were incubated with blocking serum for 1 h and then probed with the following primary antibodies overnight at 4 °C: anti-Bcl-2 (1:200), anti-Bax (1:100), (Cell Signaling Technology) and anti-MMP-2 (1:200) (Abcam, Cambridge, UK). The sections were then washed and incubated with secondary antibodies (1:200, anti-rabbit and anti-mouse; Vector Laboratories Inc., Burlingame, CA, USA) for 30 min. The sections were then stained with 3,3′-diaminobenzidine (DAB; Vector Laboratories Inc. (Burlingame, CA, USA) for 1 min and hematoxylin for 15 sec. Images were visualized and photographed using a fluorescence microscope (EVOS FL Auto 2 imaging system, Thermo Fisher Scientific).

### 4.16. Hematology and Serum Biochemical Analysis

Red blood cells (RBC), white blood cells (WBC), hemoglobin, hematocrit, mean corpuscular volume (MCV), mean corpuscular hemoglobin (MCH), MCH concentration (MCHC), red blood cell distribution width (RDW), mean platelet volume (MPV), and platelets (PLT) were analyzed with an ADVIA2120i hematology analyzer (Siemens, Munich, Germany) on whole blood. For the serum collection, blood samples were collected from the heart and separated by centrifugation at 3000 rpm for 10 min at 4 °C. ALT, AST, ALP, BUN, and creatinine were measured using an AU680 Clinical Chemistry Analyzer (Beckman Coulter, Inc., Brea, CA, USA).

### 4.17. Fingerprinting Analysis of CR

To identify the chemical constituents of CR, fingerprinting analysis was performed by LC-20A HPLC (Shimadzu Co., Kyoto, Japan) with a UV detector (PDA, 265 nm). A SunFire C18 column (250 × 4.6 mm; particle size, 5 μm; Waters Co., Milford, MA, USA) which is used for polar compound retention was maintained at 30 °C and data were acquired using LabSolutions (Shimadzu Co.) base in comparison to standards (Jatrorrhizine, Coptisine, Palmatine, Berberine).

### 4.18. Statistical Analysis

Statistical software GraphPad Prism 5.03 (GraphPad Software, Inc., La Jolla, CA, USA) was used for analysis. All experiments are presented as mean ± standard deviation (SD) and analyzed by a one-way analysis of variance (ANOVA) followed by Tukey’s post hoc test. *p*-value of <0.05 was considered statistically significant.

## 5. Conclusions

Our findings suggest that CR inhibited cell proliferation and induced apoptosis through upregulation of Bax, cleavage of PARP, and caspases, and downregulation of Bcl-2 that associated with ROS production. CR also induced autophagy by activating the expression of LC3-I/II, Atg5, Atg7, and inhibiting p62 and p-mTOR. In addition, CR inhibited cell migration though ROS production. Moreover, CR suppressed Hep3B tumor growth without toxicity. Therefore, our study provided evidence that CR represents a potential therapeutic for HCC.

## Data Availability

The data presented in this study are available within the article. Other data that support the findings of this study are available upon request from the corresponding authors.

## Supplementary Materials

The following are available online at https://www.mdpi.com/article/10.3390/ijms22094797/s1.
