# Supplementary material for: ROS-Mediated Anti-Tumor Effect of Coptidis Rhizoma against Human Hepatocellular Carcinoma Hep3B Cells and Xenografts
Source: Int J Mol Sci. 2021 Apr 30;22(9):4797. doi: 10.3390/ijms22094797 (PMC8124566; doi:10.3390/ijms22094797)
Supplement: Supplementary file 1 [file ijms-22-04797-s001.zip › ijms-1178817-supplementary/ijms-1178817-final-suppl..pdf]

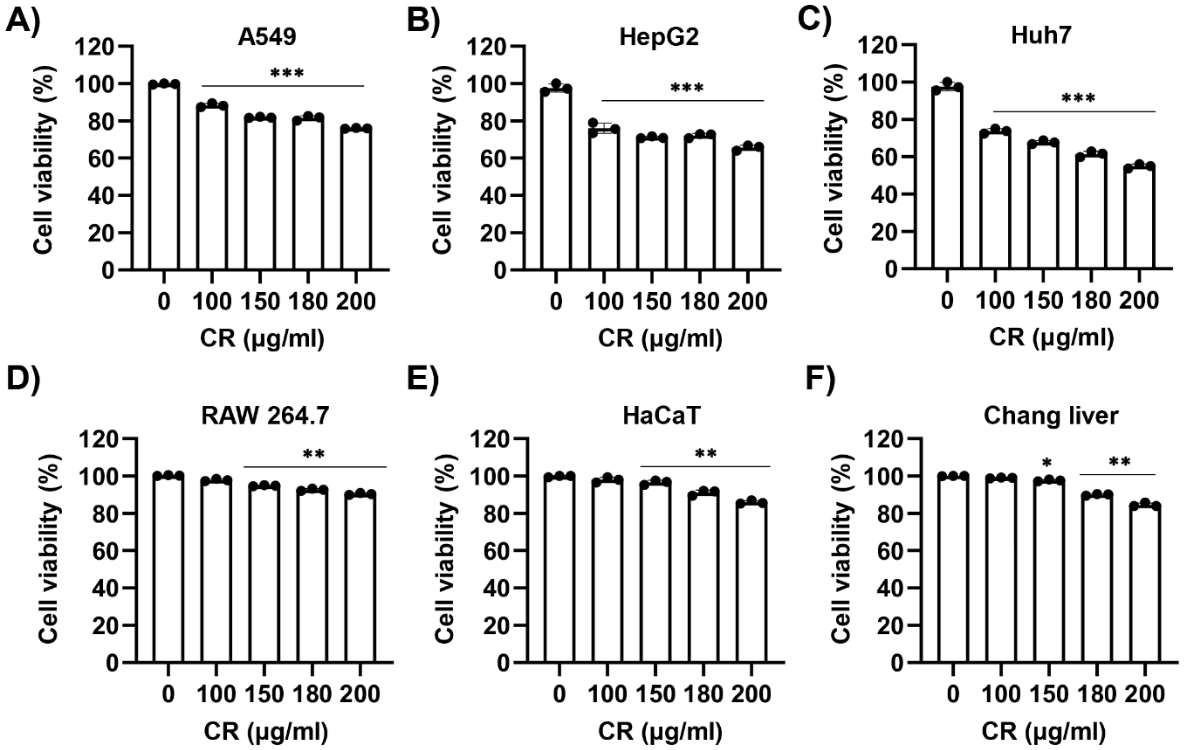

Supplementary Figure S1. Effect of Coptidis Rhizoma ethanol extract (CR) on cell viability in the various cell lines. Various concentrations of CR were treated on human lung cancer A549 cells (**A**), human hepatoma HepG2 cells (**B**), human hepatoma Huh7 cells (**C**), mouse macrophage RAW 264.7 cells (**D**), human keratinocyte HaCaT cells (**E**) and normal Chang liver cells (**F**). After 24 h, the cell viability was assessed by an MTT assay. Each bar represents the mean ± SD of three independent experiments ($n = 3$). * $p < 0.05$, ** $p < 0.01$ and *** $p < 0.001$ compared to untreated cells.

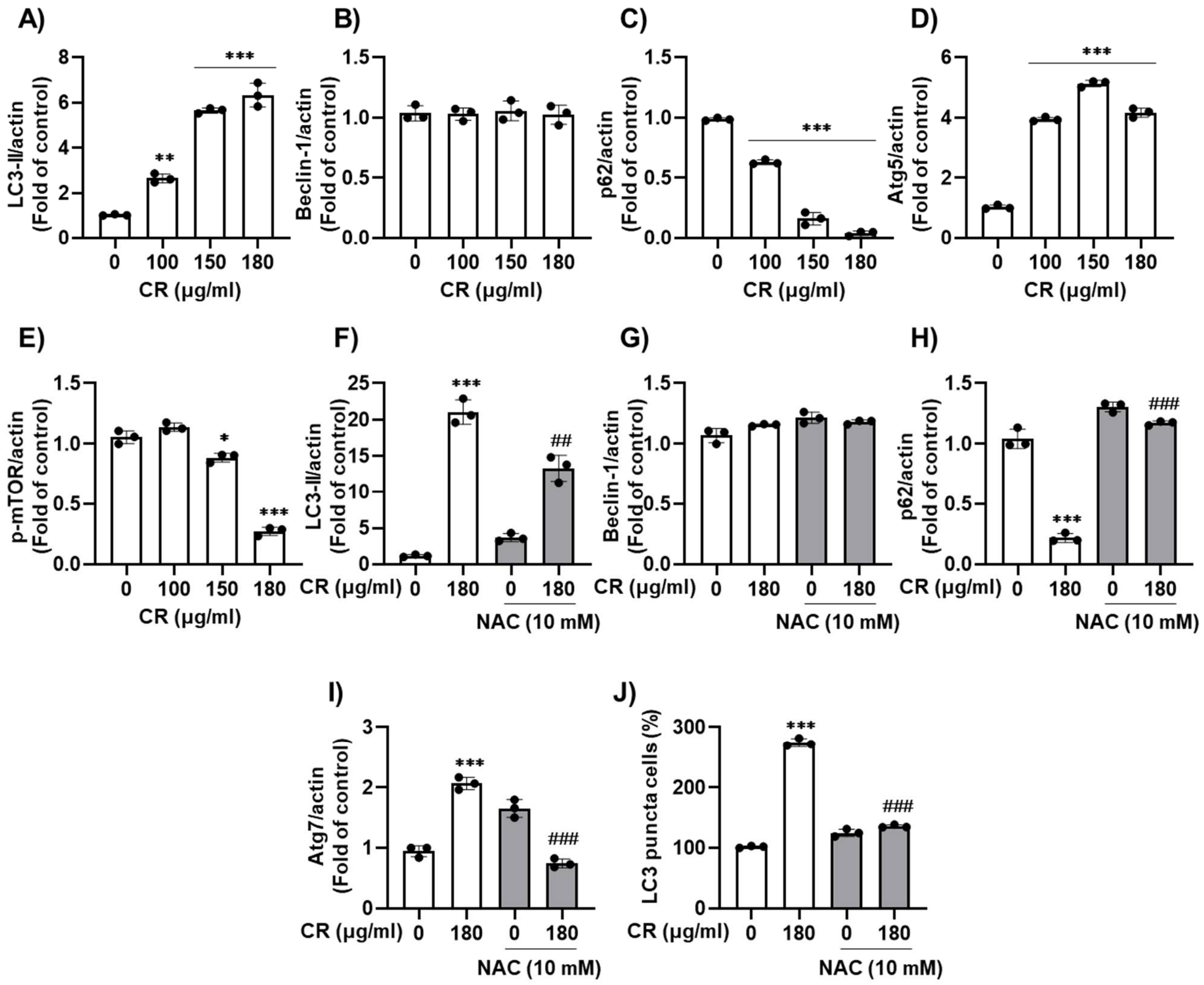

Supplementary Figure S2. Effect of Coptidis Rhizoma ethanol extract (CR) on autophagy. (**A**–**E**) Hep3B cells were treated with indicated concentration of CR for 24 h. (**F**–**I**) Hep3B cells were pre-treated with or without N-acetylcysteine (NAC), ROS inhibitor, for 1 h, and then treated with 180 μg/mL of CR for 24 h. Bar graphs indicate the relative band density in western blot analysis (n = 3). (**J**) Hep3B cells were pre-treated with or without N-acetylcysteine (NAC), ROS inhibitor, for 1 h, and then treated with 180 μg/mL of CR for 24 h. Bar graphs indicate the relative LC3 puncta in immunofluorescence (n = 3). * $p < 0.05$ and *** $p < 0.001$ compared to untreated cells; # $p < 0.05$, ## $p < 0.01$ and ### $p < 0.001$ compared to CR-treated cells.

Supplementary Table S1. The effect of oral administration of CR on organ weights in Hep3B xenograft model.

Group	Organ weights (g)				
	Heart	Lung	Liver	Kidney	Spleen
Normal	0.110 ± 0.08	0.138 ± 0.019	0.894 ± 0.111	0.258 ± 0.034	0.091 ± 0.017
Control	0.110 ± 0.015	0.139 ± 0.017	0.955 ± 0.115	0.256 ± 0.027	0.139 ± 0.042
CR 100	0.105 ± 0.008	0.132 ± 0.011	0.933 ± 0.064	0.266 ± 0.015	0.113 ± 0.026
CR 200	0.106 ± 0.011	0.140 ± 0.017	0.979 ± 0.127	0.280 ± 0.027	0.125 ± 0.043
Sorafenib	0.111 ± 0.008	0.132 ± 0.007	0.964 ± 0.152	0.296 ± 0.023	0.101 ± 0.027

After 14 days of orally administered with treatments, each organ (heart, lung, liver, kidney and spleen) was isolated from CR (*n* = 10) or sorafenib (*n* = 6), and non-treated (*n* = 10) xenograft mice as well as normal (*n* = 5) mice and measured the weight. Data are presented as means ± SD. All data showed no statistically significant difference between all groups.

Supplementary Table S2. Changes in the hematological and biochemical profiles in Hep3B xenograft model.

Parameter (units)	Group				
	Normal	Control	CR 100	CR 200	Sorafenib
RBC (× 10^3 cells/μL)	8.13 ± 0.35	8.28 ± 0.33	8.18 ± 0.43	8.60 ± 0.48	8.23 ± 0.33
WBC (× 10^3 cells/μL)	1.58 ± 0.73	1.70 ± 0.77	1.3 ± 0.78	1.64 ± 0.73	0.72 ± 0.67
Hemoglobin (g/dL)	11.58 ± 6.12	13.92 ± 0.86	13.72 ± 1.04	14.55 ± 0.79	14.48 ± 0.53
Hematocrit (%)	43.28 ± 1.85	43.63 ± 1.98	42.08 ± 2.37	44.82 ± 1.60	43.98 ± 1.90
MCV (fL)	53.20 ± 0.70	52.68 ± 1.75	51.47 ± 0.98	52.18 ± 1.99	53.48 ± 0.47
MCH (pg)	14.18 ± 7.40	16.80 ± 0.70	16.77 ± 0.69	16.95 ± 0.66	17.58 ± 0.38
MCHC (g/dL)	26.63 ± 13.91	31.88 ± 0.61	32.55 ± 0.90	32.50 ± 0.73	32.93 ± 0.92
RDW (%)	13.70 ± 0.59	13.33 ± 1.11	12.72 ± 0.31	12.88 ± 0.55	13.37 ± 0.30
MPV (fL)	10.75 ± 1.03	10.27 ± 0.40	9.95 ± 0.65	9.82 ± 0.34	9.98 ± 0.26
PLT (× 10^3 cells/μL)	804 ± 84	885 ± 73	924 ± 93	880 ± 234	827 ± 97
ALT (U/L)	101.00 ± 4.73	119.22 ± 23.20	109.28 ± 14.07	102.80 ± 13.84	126.78 ± 15.38
AST (U/L)	15.48 ± 1.10	18.97 ± 5.70	20.30 ± 4.41	20.07 ± 5.73	25.37 ± 3.00
ALP (U/L)	438.08 ± 88.15	311.37 ± 120.10	303.30 ± 66.62	335.93 ± 47.70	421.02 ± 152.86
BUN (mg/dL)	19.47 ± 2.01	18.21 ± 3.79	20.66 ± 3.12	22.96 ± 3.37	22.22 ± 2.67
Creatinine (mg/dL)	0.34 ± 0.02	0.32 ± 0.02	0.33 ± 0.03	0.32 ± 0.03	0.34 ± 0.03

After 14 days of orally administered with treatments, whole blood and serum of mice were collected from CR (n = 10), sorafenib (n = 6), non-treated (n = 10), and normal (n = 5) mice for hematological and biochemical assessment. Data are presented as means ± SD. All data showed no statistically significant difference between groups. RBC, red blood cells; WBC, white blood cells; MCV, mean corpuscular volume; MCH, mean corpuscular hemoglobin; NCHC, MCH concentration; RDW, red blood cell distribution width; MPV, mean platelet volume; PLT, platelet; ALT, alanine aminotransferase; AST, aspartate aminotransferase; ALP, alkaline phosphatase; BUN, blood urea nitrogen.
